# Gut Colonization With Carbapenem-Resistant Enterobacteriaceae in COVID-19-Recovered Patients: A Prospective Observational Study

**DOI:** 10.7759/cureus.115541

**Published:** 2026-08-31

**Authors:** Priyam Batra, Ajisha Aravindan, Priyankar K Datta, Sumit Roy Chowdhury, Rama Chaudhry, Rajeshwari Subramaniam, Nishant Verma, Anuj Singh

**Affiliations:** 1 Microbiology, All India Institute of Medical Sciences, New Delhi, New Delhi, IND; 2 Anaesthesiology, Pain Medicine and Critical Care, All India Institute of Medical Sciences, New Delhi, New Delhi, IND; 3 Anaesthesiology, All India Institute of Medical Sciences, Kalyani, Kalyani, IND

**Keywords:** antimicrobial resistance, carbapenem-resistant enterobacteriaceae, clostridioides difficile, covid-19, gut colonization, vancomycin-resistant enterococci

## Abstract

Background

The COVID-19 pandemic was associated with widespread empirical antibiotic use, raising concerns regarding disruption of gut microbiota and emergence of antimicrobial resistance. We evaluated intestinal colonization with carbapenem-resistant *Enterobacteriaceae* (CRE) among COVID-19-recovered patients and compared them with non-COVID controls.

Methods

In this prospective observational study conducted at a tertiary care center between December 2021 and March 2023, stool samples were collected from COVID-19-recovered patients who required hospitalization, COVID-19-negative controls from the community, and hospitalized COVID-19-negative controls. Samples were cultured for CRE. CRE isolates were cultured on selective media and identified using matrix-assisted laser desorption/ionization time-of-flight mass spectrometry (MALDI-TOF MS) and subsequently underwent molecular characterization for carbapenem resistance genes using the Xpert® Carba-R assay.

Results

A total of 266 participants were enrolled, comprising 87 COVID-19-recovered patients, 77 community controls, and 102 COVID-19-negative hospitalized controls. Within the COVID-19-recovered group, 20 patients (23%) had mild disease, 33 (38%) moderate, and 34 (39%) severe disease. CRE colonization was identified in 54 of 87 (62%) COVID-19-recovered patients, 50 of 77 (65%) community controls, and 56 of 102 (55%) hospitalized controls. *NDM-1* and *OXA-48* were the predominant carbapenemase genes detected among the CRE isolates.

Conclusions

Gut colonization with CRE was highly prevalent across all study groups. Hospitalization for COVID-19 was not associated with a higher prevalence of colonization compared with controls. Continued antimicrobial resistance surveillance and antibiotic stewardship remain essential in the post-pandemic era.

## Introduction

The COVID-19 pandemic resulted in unprecedented antibiotic consumption worldwide despite relatively low rates of confirmed bacterial co-infection [1]. Empirical antibiotic therapy was frequently administered because of concerns regarding secondary infections, limited diagnostic resources, and uncertainty during the early phases of the pandemic. It is estimated that approximately 75% of hospitalized COVID-19 patients received antibiotics, whereas bacterial co-infections were confirmed in only 9% of cases [2]. Furthermore, the extraordinary burden placed on healthcare systems during the pandemic compromised standard infection control practices, creating environments highly conducive to the spread of resistance. Consequently, some hospital settings observed dramatic surges in carbapenem-resistant *Enterobacteriaceae *(CRE) acquisition during the critical phases of the pandemic, with rates increasing significantly from pre-pandemic levels [3-5].

Prior use of antimicrobials strongly correlates with alterations in the gut microbiota and increased isolation of resistant pathogens. Excessive antibiotic exposure can disrupt the gut microbiome and promote colonization with multidrug-resistant organisms (MDROs), including CRE [6-8]. Colonization by these organisms has important clinical implications. CRE colonization increases the risk of subsequent invasive infection, leads to longer hospital stays, and facilitates transmission within healthcare settings [9].

Understanding the burden of post-COVID intestinal colonization with these pathogens is therefore important from both patient-care and antimicrobial-resistance perspectives. Highlighting these long-term ecological consequences is vital for prioritizing future antimicrobial stewardship efforts. This study aimed to determine the prevalence of gut colonization with CRE among COVID-19-recovered patients who required hospitalization and to compare it with two control populations.

## Materials and methods

This prospective observational study was conducted jointly by the Department of Anaesthesiology, Pain Medicine and Critical Care and the Department of Microbiology at a tertiary care center in New Delhi, India, between December 2021 and March 2023. The Institutional Ethics Committee (IEC) of All India Institute of Medical Sciences, New Delhi, India issued approval IEC-645/03.09.2021,RP-14/2021.

Adult patients (≥18 years) with laboratory-confirmed COVID-19 infection by reverse transcriptase polymerase chain reaction (RT-PCR) who required hospitalization were screened for inclusion after becoming COVID-19-negative. Patients admitted to both general wards and intensive care units (ICUs) were enrolled after obtaining informed consent from the patient or a legally authorized representative.

Patients with a prior diagnosis of gastrointestinal disease, gastrointestinal surgery, colorectal malignancy, or those who had received fecal microbiota transplantation were excluded. Inadequate or improperly handled stool samples (visible contamination with urine, collection cup leakage or cracking, or a delay of more than two hours from passage to inoculation for fresh stool samples, or 48 hours for samples stored in Cary-Blair medium) were also excluded from the final analysis.

All patients received treatment according to the prevailing institutional and national COVID-19 management guidelines during the study period. Depending on disease severity, patients received supportive care, oxygen therapy, high-flow nasal oxygen, non-invasive ventilation, or invasive mechanical ventilation, as clinically indicated.

Antimicrobial therapy was prescribed at the discretion of the treating physicians based on suspected or confirmed bacterial co-infection. Patients may also have received antiviral agents, corticosteroids, anticoagulants, and other supportive therapies as per prevailing institutional protocols.

Demographic and clinical data were collected prospectively from medical records and included age, sex, comorbidities, severity of COVID-19 illness, location of hospitalization (ward or ICU), duration of hospital stay, requirement for ventilatory support, antimicrobial exposure, corticosteroid therapy, and clinical outcomes. Details regarding antibiotic administration, including class of antibiotic, duration of therapy, number of antibiotics received, and exposure to broad-spectrum antimicrobial agents, were recorded.

Three study groups were enrolled: COVID-19-recovered cases, comprising patients admitted to the hospital with COVID-19 who recovered; control group I, comprising COVID-19-negative individuals from the community residing in the surrounding geographical area of the hospital (within the National Capital Region of Delhi, India), with no history of hospitalization and no antibiotic exposure within the preceding month; and control group II, comprising COVID-19-negative patients admitted to the hospital for other reasons.

Given the highly unpredictable nature of recruitment during the pandemic, a practical target of 100 participants per study arm was established.

Participants were asked to pass stool into a sterile collection hat. Approximately 1 g of fresh stool sample (about the size of a pea) was then collected into a sterile container using a built-in scoop in the container lid. For hospitalized patients, stool samples were collected one day before the scheduled hospital discharge or on the day of discharge. Unpreserved stool samples were sent to the laboratory for inoculation within two hours. For samples collected from the community, Cary-Blair transport medium vials were used, and samples were refrigerated at 2°C-8°C for a maximum of 48 hours. Ice packs were used for transporting samples to the laboratory.

The stool samples received were inoculated onto MacConkey agar supplemented with imipenem (1 μg/mL). Species identification was performed using matrix-assisted laser desorption/ionization time-of-flight mass spectrometry (MALDI-TOF MS). The specific carbapenemase genes in the CRE isolates were identified using the Xpert® Carba-R assay [10]. This assay detected the five major carbapenemase genes associated with carbapenem resistance, namely *blaKPC*, *blaVIM*, *blaOXA-48*, *blaIMP-1*, and *blaNDM*.

The primary objective of this study was to determine the prevalence of intestinal colonization with CRE among hospitalized COVID-19 patients. The secondary objectives were to compare CRE colonization prevalence in this cohort against two distinct control groups: community controls (to estimate the background community colonization rate) and hospitalized patients without COVID-19 (to differentiate COVID-19-specific in-hospital treatment effects from general hospitalization factors). Additionally, we aimed to characterize the specific carbapenemase genes mediating this resistance.

Data were summarized using frequencies and percentages. The chi-square test or Fisher's exact test was used, as appropriate, to analyze categorical data between groups. Microsoft Excel (2024, version 16.0; Microsoft Corporation, Redmond, WA) was used for tabulation. Figure 1 shows the cohort flowchart of subject selection and sample processing in the study.

**Figure 1:**
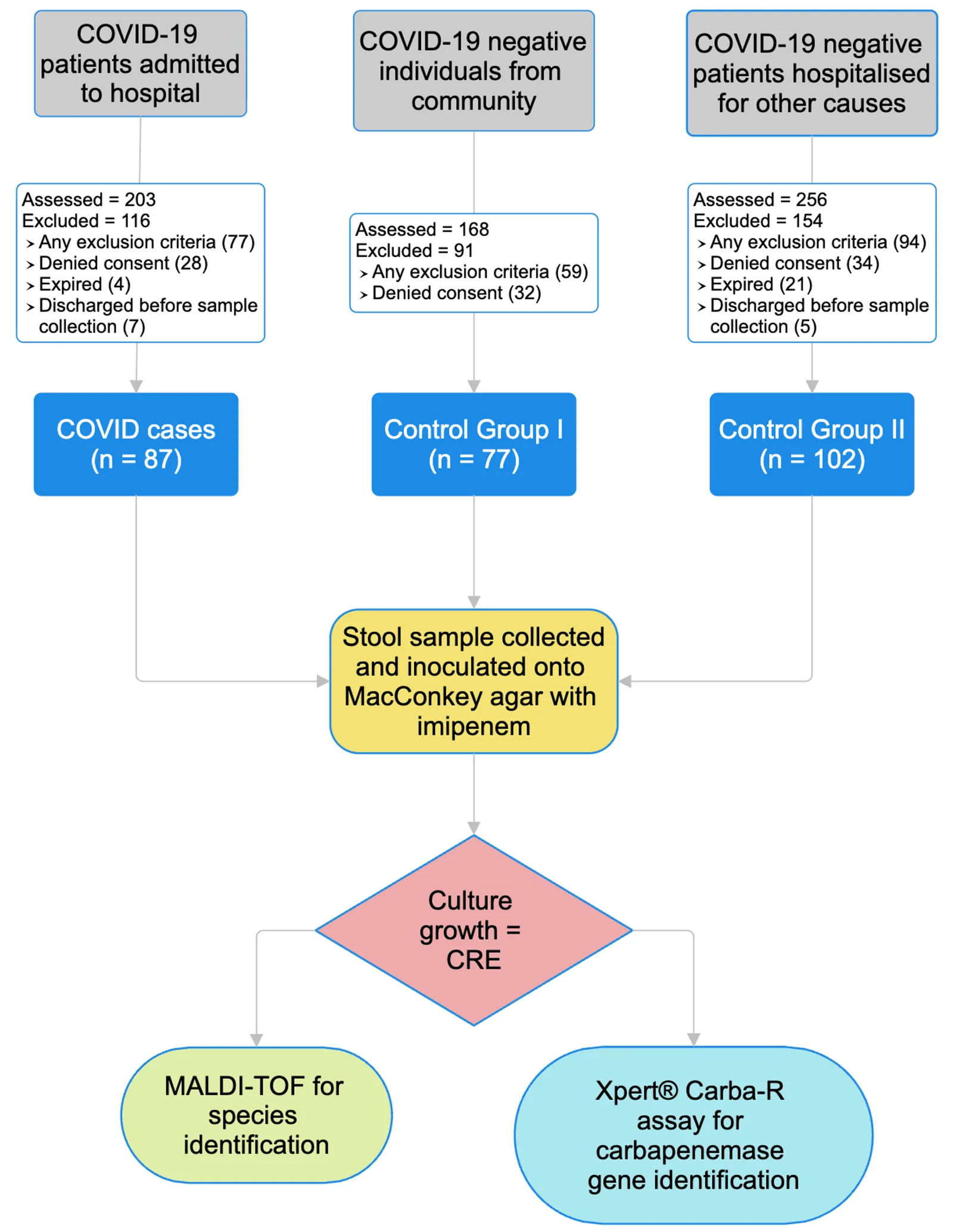
Flow diagram depicting patient recruitment process MALDI-TOF MS: Matrix-assisted laser desorption/ionization time-of-flight mass spectrometry; CRE: Carbapenem-resistant *Enterobacteriaceae*.

## Results

A total of 266 participants were enrolled in the study. The COVID-19-recovered cohort comprised 87 (33%) patients, whereas Control Group I and Control Group II included 77 (29%) and 102 (38%) participants, respectively. Within the COVID-19-recovered cohort (n = 87), disease severity was classified as mild in 20 (23%), moderate in 33 (38%), and severe in 34 (39%) patients. Notably, 61 (70%) of these patients received at least one antibacterial agent during hospitalization. The most frequently administered antibiotics were β-lactam/β-lactamase inhibitor combinations (44 (51%) patients), third-generation cephalosporins (32 (37%) patients), carbapenems (28 (32%) patients), glycopeptides (24 (28%) patients), polymyxins (12 (14%) patients), and linezolid (8 (9%) patients). Baseline characteristics of the participants are described in Table 1.

**Table 1:** Baseline characteristics of the participants COVID-19-recovered cases: patients admitted to hospital with COVID-19 who recovered; control group I: COVID-19-negative individuals from the community with no history of hospitalization or antibiotic exposure within the preceding month; control group II: COVID-19-negative patients admitted to hospital for other reasons.

Groups		COVID-19-recovered cases (n = 87)	Control group I (n = 77)	Control group II (n = 102)
Age (years), Mean (SD)		33.1 (16.9)	35.6 (15.7)	35.4 (20.7)
Sex (n%) (Male/Female)		56 (64.4%)/31 (35.6%)	57 (74%)/20 (26%)	64 (62.6%)/38 (37.4%)
Comorbidities	Diabetes mellitus, n (%)	13 (14.9%)	15 (19.4%)	21 (20.5%)
	Hypertension, n (%)	17 (19.5%)	9 (11.7%)	18 (17.6%)
Severity of COVID-19 illness (Mild/Moderate/Severe)		21 (24%)/32 (37%)/34 (39%)	NA	NA
Location of hospitalization (ward/ICU), n (%)		41 (47.1%)/46 (52.9%)	NA	49 (48%)/53 (52%)
Duration of hospital stay (days), Mean (SD)		14 (3.5)	NA	16 (5.1)

CRE colonization was detected in more than half of the participants across all study groups: in 54 of 87 (62%) COVID-19-recovered patients, 50 of 77 (65%) community controls, and 56 of 102 (55%) hospitalized controls. The distribution of CRE across the three cohorts is summarized in Table 2, and the species distribution of CRE isolates is presented in Table 3.

**Table 2:** Prevalence of carbapenem-resistant Enterobacteriaceae (CRE) COVID-19-recovered cases: patients admitted to hospital with COVID-19 who recovered; control group I: COVID-19-negative individuals from the community with no history of hospitalization in the past or antibiotic exposure within the preceding month; control group II: COVID-19-negative patients admitted to hospital for other reasons.

Organism	COVID-19-recovered cases (n = 87)	Control group I (n = 77)	Control group II (n = 102)	P-value (chi-square test)
CRE positive	54/87 (62%)	50/77 (65%)	56/102 (55%)	0.360

**Table 3:** Species distribution of carbapenem-resistant Enterobacteriaceae (CRE) isolates COVID-19-recovered cases: patients admitted to hospital with COVID-19 who recovered; control group I: COVID-19-negative individuals from the community with no history of hospitalization in the past or antibiotic exposure within the preceding month; control group II: COVID-19-negative patients admitted to hospital for other reasons.

CRE species isolated	COVID-19-recovered cases (n = 87)	Control group I (n = 77)	Control group II (n = 102)	Overall (n = 266)
Escherichia coli	32/87 (37%)	22/77 (28%)	25/102 (24.5%)	79/266 (30%)
Klebsiella pneumoniae	4/87 (5%)	9/77 (12%)	22/102 (21.5%)	35/266 (13%)
Both *E. coli* and *K. pneumoniae*	18/87 (20%)	19/77 (25%)	9/102 (9%)	46/266 (17%)
No CRE growth	33/87 (38%)	27/77 (35%)	46/102 (45%)	106/266 (40%)

Among the isolated CRE strains, *NDM-1* alone was detected in 46 (29%) samples, whereas *OXA-48* alone was detected in three (2%) samples. Co-detection of *NDM-1* and *OXA-48 *occurred in 90 (56%) samples. A triple combination of *NDM-1*, *OXA-48*, and *VIM *genes was identified in 10 (6%) samples. In the remaining 11 (7%) samples, carbapenem resistance could not be attributed to any of the specific resistance genes detectable by the GeneXpert Carba-R® assay (*IMP*, *NDM-1*, *VIM*, *KPC*, or *OXA-48*), as illustrated in Figure 2.

**Figure 2:**
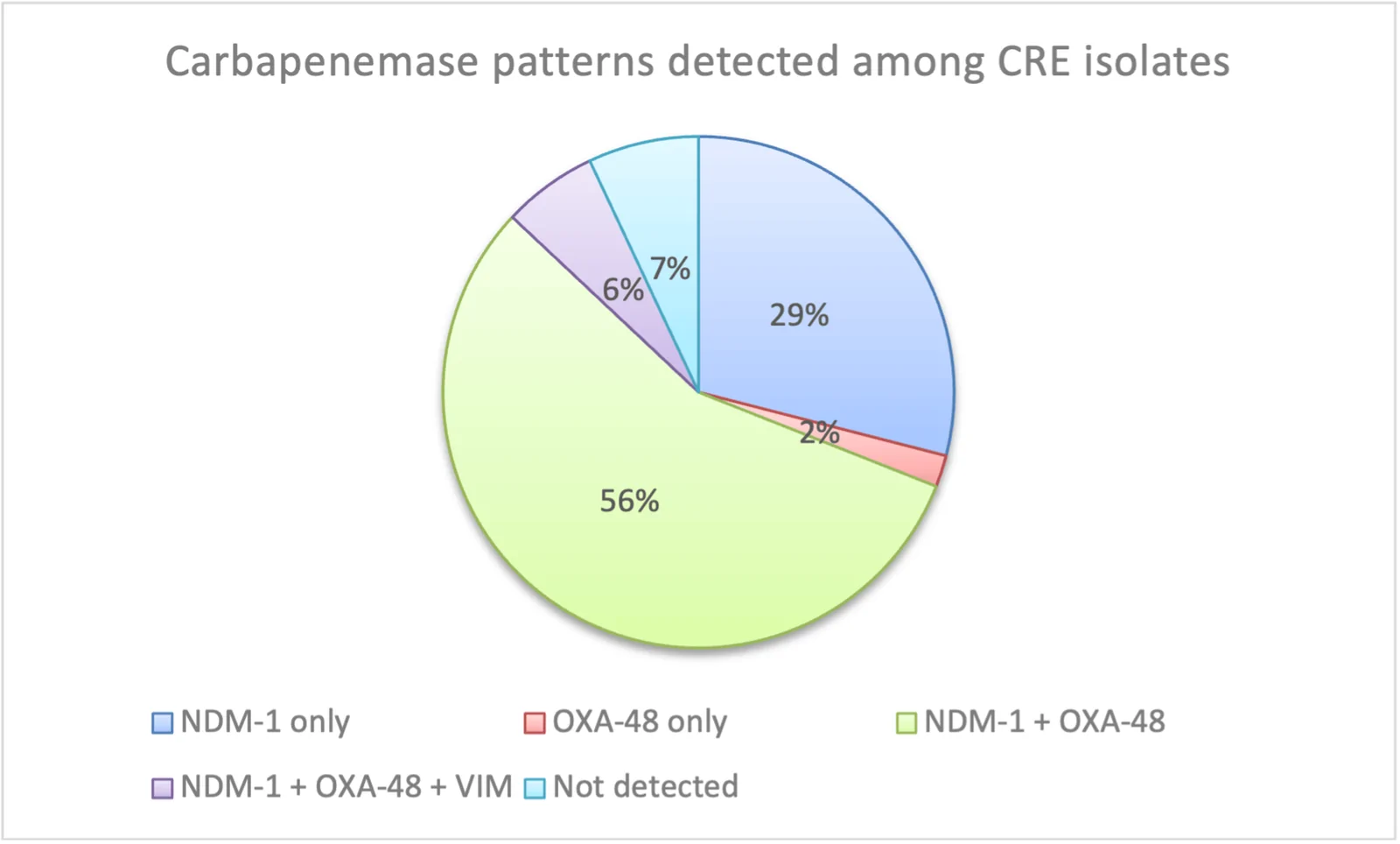
Carbapenemase patterns detected among carbapenem-resistant Enterobacteriaceae (CRE) isolates. Percentages are calculated among CRE-positive samples/isolates.

## Discussion

This prospective observational study evaluated intestinal colonization with CRE among COVID-19 recovered patients who required hospitalization and comparator populations. The principal finding was the remarkably high prevalence of CRE colonization across all study groups. More than half of the participants in each group demonstrated intestinal carriage of CRE, indicating a substantial reservoir of antimicrobial-resistant organisms within both healthcare and community settings. However, hospitalization due to COVID-19 infection was not associated with a significantly greater prevalence of CRE colonization compared with community or non-COVID hospitalized controls.

CRE colonization was detected in more than half of the study participants. These findings are noteworthy because intestinal colonization serves as an important reservoir for subsequent infection and transmission of resistant organisms. The uniformly high prevalence observed across all study groups suggests that antimicrobial-resistant organisms may be widely disseminated within both healthcare facilities and the community.

Several studies have reported an increase in antimicrobial-resistant organism colonization and infection during the COVID-19 pandemic. A systematic review found an overall increase in CRE infection or colonization ranging from 6.9% to 314.8%, with most studies demonstrating an increase in carbapenem-resistant *K. pneumoniae* infections [5]. These observations have largely been attributed to widespread antibiotic exposure, prolonged hospitalization, increased use of invasive devices, and corticosteroid therapy during the pandemic.

Despite these observations, our study did not demonstrate a higher prevalence of CRE colonization among COVID-19 recovered patients compared with controls. One possible explanation is that antimicrobial-resistant organisms have become widely established in both healthcare and community settings toward the end of the pandemic period, thereby reducing detectable differences between populations. Additionally, colonization acquired during acute COVID-19 illness may not persist indefinitely, and recovery-associated restoration of gut microbiota could potentially diminish long-term carriage in some individuals. The absence of significant differences between groups suggests that antimicrobial resistance colonization may represent a broader public health challenge rather than a phenomenon confined to COVID-19 survivors.

Molecular characterization of CRE isolates revealed a predominance of *NDM-1* and *OXA-48* carbapenemase genes. These findings are consistent with previously reported resistance mechanisms in the Indian subcontinent and reinforce the continuing challenge posed by these highly transmissible carbapenemase determinants [11-13]. The persistence of these resistance genes among colonizing organisms highlights the importance of continued surveillance and antimicrobial stewardship efforts aimed at limiting further dissemination of carbapenem-resistant pathogens. It is important to note that in approximately 7% of CRE isolates, common carbapenemase genes could not be identified. In these isolates, resistance may have been mediated by alternative mechanisms such as porin loss, efflux pump overexpression, or other carbapenemase genes.

The present study had some limitations. It was conducted at a single, tertiary-care center, which restricts the generalizability of its findings. The observational study design precludes the establishment of a causal relationship between prior COVID-19 infection and antimicrobial-resistant colonization. Absence of microbiological data from the baseline stool samples limits the ability to determine whether CRE colonization was acquired during the hospitalization or was already present. As participant recruitment for each of the three groups occurred simultaneously and independently, matching cases with controls was not possible. Finally, due to the constrained sample size, detailed multivariate analyses examining the relationships among disease severity, illness duration, antibiotic exposure, corticosteroid use, hospitalization characteristics, and the risk of resistant organism colonization were not performed. Such analyses might have provided deeper insights into the specific confounding factors influencing colonization patterns.

## Conclusions

This study demonstrates a substantial burden of intestinal colonization with CRE in both community and hospitalized populations, irrespective of prior COVID-19 infection status. Although recovery from COVID-19 was not associated with an increased prevalence of colonization, the consistently high rates of CRE carriage observed across all study groups suggest that antimicrobial resistance has become deeply entrenched within local healthcare and community ecosystems. The predominance of *NDM-1* and *OXA-48* carbapenemase genes further highlights the ongoing challenge posed by highly resistant *Enterobacteriaceae *in India. Consequently, these findings underscore an urgent need to shift focus from COVID-19-specific concerns toward broader, sustained antimicrobial stewardship. Implementing active surveillance for resistant organism carriage and enhancing infection prevention strategies are critical to limiting transmission and reducing the future burden of difficult-to-treat infections.
